# Preparation and characterization of ZnO microcantilever for nanoactuation

**DOI:** 10.1186/1556-276X-7-176

**Published:** 2012-03-08

**Authors:** Peihong Wang, Hejun Du, Shengnan Shen, Mingsheng Zhang, Bo Liu

**Affiliations:** 1 School of Mechanical and Aerospace Engineering, Nanyang Technological University, 50 Nanyang Avenue, Singapore, 639798, Republic of Singapore; 2 Data Storage Institute, 5 Engineering Drive 1, Singapore, 117608, Republic of Singapore

**Keywords:** ZnO thin film, piezoelectric cantilever, micromachining technique, transverse piezoelectric constant

## Abstract

Zinc oxide [ZnO] thin films are deposited using a radiofrequency magnetron sputtering method under room temperature. Its crystalline quality, surface morphology, and composition purity are characterized by X-ray diffraction [XRD], atomic force microscopy [AFM], field-emission scanning electron microscopy [FE-SEM], and energy-dispersive X-ray spectroscopy [EDS]. XRD pattern of the ZnO thin film shows that it has a high c-axis-preferring orientation, which is confirmed by a FE-SEM cross-sectional image of the film. The EDS analysis indicates that only Zn and O elements are contained in the ZnO film. The AFM image shows that the film's surface is very smooth and dense, and the surface roughness is 5.899 nm. The microcantilever (Au/Ti/ZnO/Au/Ti/SiO_2_/Si) based on the ZnO thin film is fabricated by micromachining techniques. The dynamic characterizations of the cantilever using a laser Doppler vibrometer show that the amplitude of the cantilever tip is linear with the driving voltage, and the amplitude of this microcantilever's tip increased from 2.1 to 13.6 nm when the driving voltage increased from 0.05 to 0.3 V_rms_. The calculated transverse piezoelectric constant *d*_31 _of the ZnO thin film is -3.27 pC/N. This *d*_31 _is high compared with other published results. This ZnO thin film will be used in smart slider in hard disk drives to do nanoactuation in the future.

## Introduction

As the recording density of hard disk drive [HDD] further increases, the spacing between the slider and the disk decreases quickly. A 10-Tb/in^2 ^recording density requires a 2 to 3 nm or even less of head-media spacing when HDD is working [1]. In that case, contact between slider and disk will not be avoided completely. So, it is necessary to detect the slider-disk contact and then adjust the spacing to decrease the damage to the head-disk interface. Due to quick response and contact detection ability, many piezoelectric sensors/actuators based on bulk and thin film lead zirconate titanate [PZT] have been proposed in order to solve the above problem [2,3]. However, it is hard to fabricate a bulk PZT material into a microscale dimension. Meanwhile, the deposition of PZT thin film usually requires processing at over 600°C [4], which is not compatible with fabrication of magnetic heads in HDD technology. Compared to PZT material, ZnO thin film also has good piezoelectric quality, and its microfabrication does not need head process under very high temperature [5]. So, ZnO film-based piezoelectric sensors/actuators are being designed and used in smart slider to detect the head-disk contact and then adjust the flying height in our ongoing project.

There were many papers about the fabrication and characterization of ZnO films in the past decades [6-8]. However, few reports presented the piezoelectric quality of ZnO film quantitatively. In this paper, a ZnO thin film for the application of nanoactuator was deposited using a radiofrequency [RF] magnetron sputtering system under room temperature and was characterized by X-ray diffraction [XRD], energy-dispersive X-ray spectroscopy [EDS], field-emission scanning electron microscopy [FE-SEM], and atomic force microscopy [AFM]. Moreover, a ZnO film-based piezoelectric microcantilever was fabricated by micromachining techniques. The dynamic response of the piezoelectric cantilever was measured using a laser Doppler vibrometer [LDV]. The calculation result showed that the transverse piezoelectric constant *d*_31 _of the ZnO thin film is -3.27 pC/N. This *d*_31 _is high compared with other published results. In the future, this ZnO thin film will be used in smart slider to do nanoactuation in HDDs.

### Experimental details

ZnO thin films were deposited by RF magnetron sputtering system using a ZnO target (99.99%) with a diameter of 2 in. and a thickness of 3 mm. The substrate comprised p-type silicon (100) with a SiO_2 _layer and a Au/Ti seed layer. The thickness of the SiO_2 _layer and the Au/Ti seed layer were 1 μm and 300 nm/50 nm, respectively. The chamber was down to 5 × 10^-6 ^Torr using a molecular pump before introducing mixed Ar and O_2 _gases. In the deposition of the ZnO thin film, the RF power was 70 W, the working pressure was 0.8 Pa, the O_2_/(Ar + O_2_) gas ratio was 0.25, and the substrate temperature was at room temperature. The above deposition condition was from our previous optimization result. The corresponding deposition rate of the ZnO film was about 0.28 μm/h, and the deposition time was 4.5 h.

The ZnO piezoelectric microcantilever was fabricated by micromachining techniques. The substrate was (100) p-type silicon wafer on which a 1 μm of silicon dioxide [SiO_2_] was grown using a thermal oxidation method. Both the top and bottom electrodes were Au/Ti which was more suitable than the Al electrode used in our previous fabrication. The microfabrication process of the cantilever mainly included ZnO thin film etching, top and bottom electrode deposition and patterning, silicon DRIE etching, and finally the microcantilever's release. The detailed steps are the following: (1) Au/Ti bottom electrode is sputtered on the SiO_2 _surface and patterned by lift-off technique; (2) ZnO thin film is sputtered on the bottom electrode; (3) Au/Ti top electrode is sputtered on the ZnO film surface and patterned by lift-off technique; (4) ZnO film is wet etched by a diluted HCl solution to expose the bottom electrode and the SiO_2 _layer around the cantilever; (5) the SiO_2 _layer is etched by reactive-ion etching technique, and then the Si layer below with a specific thickness is etched by deep reactive-ion etching technique; and (6) finally, the SiO_2 _layer and the Si substrate are etched through to release the cantilever from the backside of the wafer.

The crystalline structure of the ZnO film was examined using an X-ray diffractometer (PW 1830, Philips, Singapore, Republic of Singapore). The chemical composition and the stoichiometry of the ZnO films were analyzed by EDS equipped with a scanning electron microscope (JSM-5600 V, JEOL, Singapore, Republic of Singapore). The surface morphology of the ZnO film was measured by AFM (3000 SPM, DI, Santa Barbara, CA, USA). The multilayered cross sections of the ZnO film were observed by FE-SEM (JSM-7600F, JEOL). The dynamic characteristics of the ZnO piezoelectric microcantilever were tested using a LDV (PSV300, Polytec, Irvine, CA, USA).

## Results and discussion

**Figure 1:**
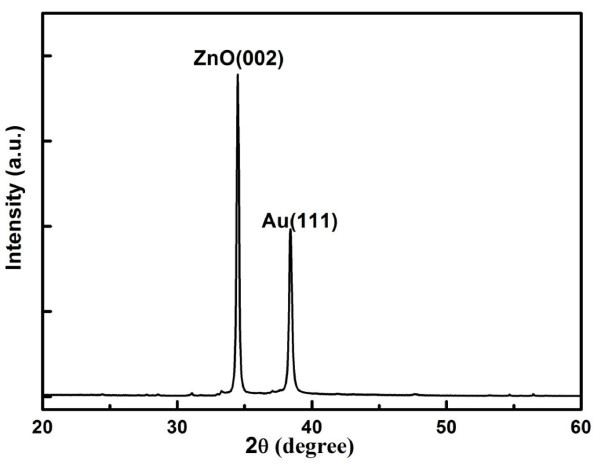
**XRD pattern of the ZnO thin film**.

The XRD pattern of the ZnO thin film shown in Figure 1 indicates that the diffraction peak located around 34.422° is very high compared with other peaks and is just the ZnO(002) diffraction peak. So, the ZnO thin film on the Au/Ti bottom electrode has a highly c-axis-preferred orientation, which is necessary to achieve a ZnO film with high piezoelectric quality. The full width at half maximum [FWHM] of the ZnO(002) diffraction peak can be obtained from the XRD pattern, and it is 0.198° for the deposited ZnO film. The grain size in the ZnO thin film can be calculated using the Debye-Scherrer formula: $D=\frac{0.89\lambda }{B\text{cos}\theta }$, where *λ, B*, and *θ *were the wavelength of the X-ray source (*λ *is equal to 0.154056 nm for the Cu target), the FWHM of the ZnO(002) diffraction peak, and the Bragg diffraction angle, respectively. The calculated grain size in the ZnO thin film is 41.566 nm.

**Figure 2:**
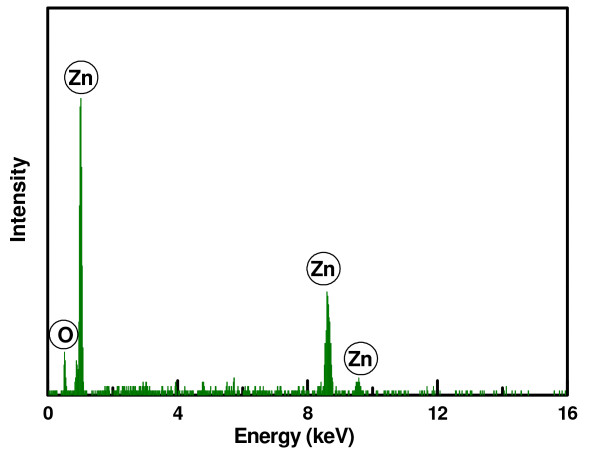
**EDS analysis of the ZnO thin film**.

To further confirm the chemical composition of the ZnO film, an EDS analysis shown in Figure 2 was done. It reveals that only Zn and O elements were contained in the ZnO thin film. Compositional analysis of the EDS spectrum indicated that the atomic ratio of Zn/O in the ZnO film is 47.90:52.10. This means that the ZnO film has a little excessive oxygen, which is maybe from the oxygen ions that existed on the surface.

**Figure 3:**
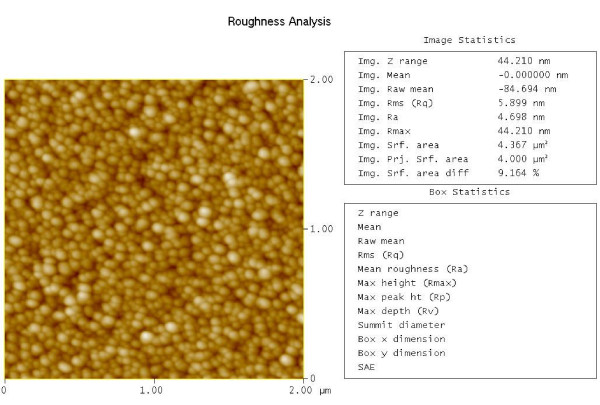
**AFM image and roughness analysis of the ZnO thin film**.

Figure 3 shows the 2-D AFM image and the roughness analysis of the ZnO thin film. As can be seen in Figure 3, the grain sizes in the ZnO thin film are very uniform, and the surface is very dense and smooth. The roughness analysis from the AFM showed that the surface roughness (RMS value) is just 5.899 nm.

**Figure 4:**
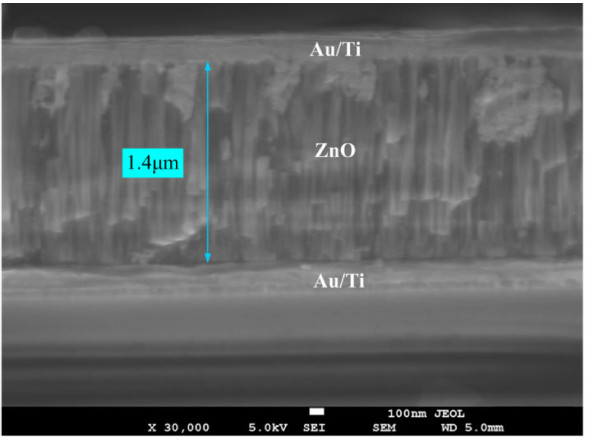
**The cross-sectional FE-SEM image of the ZnO thin film with top and bottom electrodes**.

**Figure 5:**
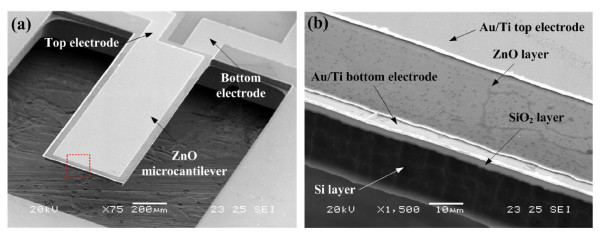
**SEM images of the fabricated ZnO microcantilever chip**. (**a**) Entire view of the microcantilever, and (**b**) enlarged view of the multilayered structure marked in (a).

The cross-sectional morphology of the multilayer structure of the ZnO microcantilever was observed by FE-SEM and is shown in Figure 4. It clearly shows that the ZnO thin film in the cantilever is polycrystalline. The columnar texture of the ZnO thin film can be seen in Figure 4, which indicates that the ZnO film is highly c-axis-oriented and so has hexagonal crystals. It also shows that the thickness of the ZnO film is about 1.4 μm. The SEM images of the fully fabricated ZnO cantilever and the enlarged multilayered structure of the cantilever are shown in Figure 5. It shows clearly that the microcantilever stably maintained its freestanding state without structural deformations. The dimension of the Si cantilever is about 1, 500 × 500 × 24 μm^3^.

**Figure 6:**
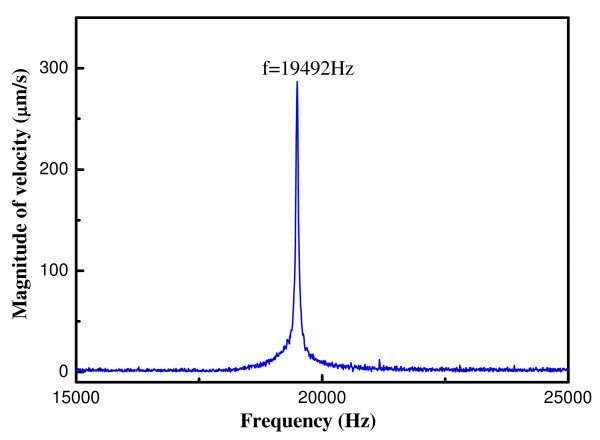
**The magnitude of velocity of cantilever tip versus frequency of input vibration**.

The above characterizations of the ZnO thin film and the ZnO microcantilever were just done qualitatively. Actually, the most important issue for the ZnO piezoelectric film is having high piezoelectric performance. So in the following experiment, LDV was used to measure the dynamic response of the ZnO microcantilever first, and then the transverse piezoelectric constant *d*_31 _of the ZnO film was calculated using the tested data from LDV. The relationship between the magnitude of velocity of cantilever's tip and the frequency of input vibration is given in Figure 6. It clearly shows that the natural frequency of the microcantilever is 19, 492 Hz.

**Figure 7:**
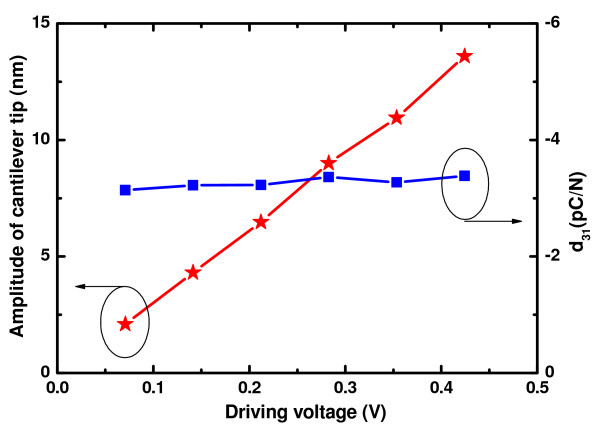
**Measured amplitude of cantilever tip and *d*_31 _of ZnO film versus applied voltage on microcantilever**. During the experiment, the frequency of the AC driving voltage is 1 kHz.

When an AC voltage signal with sine waveform was applied on the top and bottom electrodes of the ZnO microcantilever, the cantilever generated vibration according to piezoelectric effect. Then, the LDV can detect the vibration of the cantilever and measure the velocity and displacement of the cantilever. The measured amplitude of the cantilever tip versus the driving voltage is given in Figure 7. In this experiment, the frequency of the driving voltage is fixed at 1 kHz. As can be seen from Figure 7, the amplitude of the ZnO cantilever tip increased from 2.1 to 13.6 nm when the driving voltage increases from 0.05 to 0.3 V_rms_. Moreover, the amplitude of the cantilever tip increases linearly with the driving voltage.

For a given piezoelectric heterogeneous bimorph including one elastic layer and one piezoelectric layer, both of which have same width, the deflection of the cantilever can be expressed as [9]:

(1) $$\delta \left(x\right)=\frac{3x^{2}t_{\text{e}}\left(t_{\text{e}}+t_{\text{p}}\right)E_{\text{e}}E_{\text{p}}Vd_{31}}{E_{\text{e}}^{2}t_{\text{e}}^{\text{4}}+E_{\text{e}}E_{\text{p}}\left(4t_{\text{e}}^{3}t_{\text{p}}+6t_{\text{e}}^{2}t_{\text{p}}^{\text{2}}+4t_{\text{e}}^{}t_{\text{p}}^{\text{3}}\right)+E_{\text{p}}^{2}t_{\text{p}}^{4}},$$

where *x *is the distance from the fixed end of the cantilever; *δ(x) *is the deflection; *t*_p _and *t*_e _are the thicknesses of piezoelectric and elastic layers (namely Si substrate), respectively; *E*_p _and *E*_e _are Young's modulus of piezoelectric and elastic materials, respectively; *V *is the applied voltage; and *d*_31 _is the transverse piezoelectric constant of the piezoelectric material. For our fabricated ZnO microcantilever, all the parameters except *d*_31 _are known. *x *is the length of the cantilever which is 1, 500 μm, *t*_p _is 1.4 μm, *t*_e _is 24 μm, *E*_p _is 1.8 × 10^11 ^Pa, and *E*_e _is 1.9 × 10^11 ^Pa. *δ(x) *and *V *can be obtained from Figure 7. So, the *d*_31 _of the ZnO thin film can be calculated using Equation 1. The calculated *d*_31 _of the ZnO film versus different driving voltages is given in Figure 7. As can be seen from Figure 7, the transverse piezoelectric constant *d*_31 _of the ZnO film is almost changeless under different driving voltages. The average value of *d*_31 _can be calculated as -3.27 pC/N which has the same order of magnitude as the -5.43 pC/N for the ZnO bulk material [10].

Although the transverse piezoelectric constant *d*_31 _of our deposited ZnO film is smaller than that of the ZnO bulk material, it is high compared with other results [9,11-13]. Moreover, many ZnO films suffered heating or post-annealing process in the experiments, but in our case, the deposition of ZnO film was under room temperature. So, the piezoelectric quality of the ZnO film will be surely increased further if heating or post-annealing process is carried out on our fabricated ZnO thin film. The relative experiment is ongoing.

## Conclusion

ZnO piezoelectric microcantilevers were fabricated and characterized in this paper. The ZnO film was deposited on Au/Ti bottom electrode using RF magnetron sputtering system under room temperature. XRD pattern showed that the ZnO film has a highly c-axis-preferred orientation, and the grain size in the ZnO film was 41.566 nm. The EDS spectrum indicated that only Zn and O elements were contained in the ZnO film. The AFM image showed that the surface of the ZnO film was very uniform, and the surface roughness was 5.899 nm. The FE-SEM cross-sectional image of the ZnO film confirmed that the ZnO film grows columnar, and the direction is perpendicular to the surface. The piezoelectric microcantilever based on the ZnO film was fabricated by micromachining techniques. Its dimension is about 1, 500 × 500 × 25 μm^3^. The dynamic characterization of the microcantilever using LDV showed that the deflection of the cantilever increases with the driving voltage linearly, and the experimental result indicated that the amplitude of the ZnO cantilever tip increased from 2.1 to 13.6 nm when the driving voltage increased from 0.05 to 0.3 V_rms_. The transverse piezoelectric constant *d*_31 _of the ZnO film calculated using LDV data is -3.27 pC/N which is high compared with other published results. This ZnO thin film will be used in our ongoing design, simulation, and fabrication of ZnO film-based smart slider in hard disk drives.

## Competing interests

The authors declare that they have no competing interests.

## Authors' contributions

PW carried out the preparation of the ZnO thin films, participated in the characterization of the ZnO thin films, and drafted the manuscript. HD conceived the study and participated in its design partially. SS participated in the characterization of the ZnO thin films. MZ participated in the characterization of the ZnO thin films and the coordination. BL conceived the study partially and participated in its coordination. All authors read and approved the final manuscript.
